# Supplementary material for: Thermochemical Study of 1-Methylhydantoin
Source: Molecules. 2022 Jan 16;27(2):556. doi: 10.3390/molecules27020556 (PMC8777693; doi:10.3390/molecules27020556)
Supplement: Supplementary file 1 [file molecules-27-00556-s001.zip › molecules-1463411-SI.pdf]

Support information for:

Thermochemical study of 1-methylhydantoin

J. Manuel Ledo[1], Henoc Flores[2,*], Fernando Ramos[2,*], E. Adriana Camarillo

[1] Complejo Regional Mixteca, Campus Izúcar de Matamoros. Benemérita Universidad Autónoma de Puebla. Carr. Atlixco - Izúcar de Matamoros 141, C.P. 74570 San Martín Alchichica, Izúcar de Matamoros, Pue, México.
[2] Facultad de Ciencias Químicas, Benemérita Universidad Autónoma de Puebla. 14 sur y Av. San Claudio, C.P. 72570, Puebla Pue, México.

*Corresponding authors. e–mail: henoc.flores@correo.buap.mx
fernando_.ramos@correo.buap.mx

CONTENT **Page**

Table S1. Fraction purity, fusion temperature and fusion enthalpies of 1MH, obtained by DSC determinations, at $p°$=0.1 MPa. **S3**

Table S2. Results of combustion experiments of 1-methylhydantoin, at T = 298.15 K and p° = 0.1 MPa. **S4**

Table S3. Complete series of thermogravimetric experiments of 1MH. **S5**

Table S1. Fraction purity, fusion temperature and fusion enthalpy of 1MH obtained by DSC determinations, at $p°$=0.1 MPa

m_{sample}[a] / mg	purity / mole fraction	T_{fus} / K	$\Delta_{fus}H_m(T_{fus})$ / kJ·mol^{-1}
	1-methylhydantoin (1MH)		
4.154	0.9995	431.0	21.39
2.224	0.9994	431.1	22.14
2.458	0.9995	431.0	22.43
3.033	0.9996	430.7	22.12
	0.9995 ± 0.0002 [b]	431.0 ± 0.5 [b]	22.02 ± 1.11 [b]

[a] Standard uncertainty $u(m_{sample})$=0.0001 mg, standard uncertainties u is $u(p)$=1 kPa

[b] The uncertainties are expanded ones: coverage factor k=2.45 and 0.95 level of confidence for a t-student distribution, these include contributions from the DSC calibration.

Table S2. Results of combustion experiments of 1-methylhydantoin, at T = 298.15 K and $p°$ = 0.1 MPa[a]

m (cpd)/g	0.99985	1.00448	1.00726	1.00841	0.99023	1.00541	1.00807
m (po)/g	0.00000	0.10818	0.09829	0.10419	0.11018	0.10279	0.12596
m (cotton)/g	0.00241	0.00228	0.00214	0.00226	0.00239	0.00203	0.00203
m (Pt)/g	11.49247	11.49461	11.49692	11.49592	11.49847	11.49623	11.49719
T_i/K	295.4843	295.4937	295.4973	295.4965	295.4964	295.4997	295.4973
T_f/K	297.2506	297.7597	297.7223	297.7504	297.745	297.7425	297.8456
ΔT_{corr}/K	0.0492	0.0462	0.0462	0.0459	0.0459	0.0467	0.0442
ΔT_c /K	1.7171	2.2198	2.1788	2.208	2.2027	2.1961	2.3041
ε_i(cont)/kJ·K^{-1}	0.0168	0.0171	0.0171	0.0171	0.0171	0.0171	0.0171
ε_f(cont)/kJ·K^{-1}	0.0185	0.0191	0.0191	0.0191	0.0191	0.0191	0.0192
-ΔU_{IBP}/kJ	17.4052	22.5039	22.0881	22.3842	22.3305	22.2636	23.3589
$\Delta U(HNO_3)$/kJ	0.0733	0.0825	0.0855	0.0855	0.0764	0.0794	0.0794
ΔU_{ign}/kJ	0.0042	0.0042	0.0042	0.0042	0.0042	0.0042	0.0042
ΔU_Σ/kJ	0.0138	0.0154	0.0153	0.0154	0.0153	0.0154	0.0158
(-$m\Delta_c u°$) (po)/kJ	0.0000	5.0018	4.5445	4.8176	5.0944	4.7528	5.8243
(-$m\Delta_c u°$) (cotton)/kJ	0.0409	0.0387	0.0363	0.0383	0.0405	0.0344	0.0344
(-$\Delta_c u°$) (cpd)/kJ·g^{-1}	17.2798	17.2880	17.2810	17.2821	17.2727	17.2881	17.2657

⟨-$\Delta_c u°$ (298.15 K)/kJ·g^{-1}⟩ = 17.2796 ± 0.0031

[a] Here m (cpd), m (po), m (cotton), and m (Pt) are the masses of the compound, paraffin oil, cotton thread, and platinum (which includes masses of both the crucible and the ignition wire), respectively. ΔT_c is the corrected temperature rise, ε_i(cont) and ε_f(cont) are the energy equivalents of the bomb contents in the initial and final states. $\Delta U(HNO_3)$ is the energy correction for the nitric acid formation, ΔU_{ign} is the ignition energy, ΔU_{IBP} is the energy of the isothermal bomb process (calculated as $\Delta U_{\mathrm{IBP}} = \varepsilon(\mathrm{calor}) \cdot (-\Delta T_{\mathrm{c}}) + \varepsilon_{\mathrm{i}}(\mathrm{cont}) \cdot (T_{\mathrm{i}} - 298.15\ \mathrm{K}) + \varepsilon_{\mathrm{f}}(\mathrm{cont}) \cdot (298.15\ \mathrm{K} - T_{\mathrm{f}} + \Delta T_{\mathrm{corr}}) + \Delta U_{\mathrm{ign}}$). ΔU_Σ is the correction to standard states, and $\Delta_c u°$ (cpd) is the mass energy of combustion of compound (calculated as $\Delta_{\mathrm{c}} u^{\circ}(\mathrm{cpd}) = [\Delta U_{\mathrm{IBP}} + \Delta U_{\Sigma} - m\Delta_{\mathrm{c}} u^{\circ}(\mathrm{cotton}) - m\Delta_{\mathrm{c}} u^{\circ}(\mathrm{auxiliar})]/m(\mathrm{cpd})$). The uncertainties attached to averages of specific combustion energies are the standard deviations of mean, *i.e.* they are standard uncertainties.

Table S3. Complete Series of thermogravimetric experiments of 1MH

$\frac{T}{\text{K}}$	$\frac{m}{\text{mg}}$	$\frac{(\text{d}m/\text{d}t)\cdot 10^9}{\text{kg}\cdot\text{s}^{-1}}$	$\frac{(1/T)\cdot 10^3}{\text{K}^{-1}}$	ln(dm/dt ·T)
		Series 1		
440.0	15.9006	0.0149	2.273	-18.846
442.0	15.7015	0.0161	2.262	-18.759
444.0	15.4900	0.0174	2.252	-18.678
446.0	15.2653	0.0188	2.242	-18.594
448.0	15.0273	0.0204	2.232	-18.512
450.0	14.7724	0.0220	2.222	-18.431
452.0	14.4989	0.0237	2.212	-18.351
454.0	14.2039	0.0255	2.203	-18.274
456.0	13.8841	0.0274	2.193	-18.197
458.0	13.5428	0.0295	2.183	-18.120
460.0	13.1747	0.0317	2.174	-18.043
		Series 2		
440.0	14.5161	0.0147	2.273	-18.853
442.0	14.3188	0.0160	2.262	-18.765
444.0	14.1089	0.0174	2.252	-18.676
446.0	13.8855	0.0189	2.242	-18.591
448.0	13.6482	0.0204	2.232	-18.509
450.0	13.3932	0.0221	2.222	-18.428
452.0	13.1196	0.0238	2.212	-18.349
454.0	12.8250	0.0256	2.203	-18.272
456.0	12.5055	0.0275	2.193	-18.195
458.0	12.1640	0.0295	2.183	-18.119
460.0	11.7969	0.0316	2.174	-18.046
		Series 3		
440.0	13.7715	0.0143	2.273	-18.886
442.0	13.5814	0.0154	2.262	-18.805
444.0	13.3780	0.0167	2.252	-18.718
446.0	13.1624	0.0180	2.242	-18.638
448.0	12.9317	0.0196	2.232	-18.548
450.0	12.6857	0.0212	2.222	-18.466
452.0	12.4210	0.0229	2.212	-18.385
454.0	12.1358	0.0246	2.203	-18.309
456.0	11.8282	0.0265	2.193	-18.231
458.0	11.4976	0.0284	2.183	-18.156
460.0	11.1420	0.0306	2.174	-18.078
		Series 4		
440.0	13.7038	0.0151	2.273	-18.832
442.0	13.5037	0.0163	2.262	-18.748
444.0	13.2910	0.0176	2.252	-18.665
446.0	13.0660	0.0191	2.242	-18.581
448.0	12.8254	0.0206	2.232	-18.501
450.0	12.5668	0.0222	2.222	-18.421
452.0	12.2899	0.0240	2.212	-18.340
454.0	11.9936	0.0257	2.203	-18.265
456.0	11.6734	0.0277	2.193	-18.188
458.0	11.3278	0.0297	2.183	-18.114
460.0	10.9575	0.0318	2.174	-18.039

Series 1 ln(dm/dt T)=-0.4–8110.9/T; r^2=0.9999; σ_a=0.1; σ_b=17.9; $\Delta_l^g H_m$(450.0 K)/kJ·mol^{-1}=67.4 ± 0.1

Series 2 ln(dm/dt T)=-0.3–8156.2/T; r^2=0.9997; σ_a=0.1; σ_b=48.7; $\Delta_l^g H_m$(450.0 K)/kJ·mol^{-1}=67.8 ± 0.4

Series 3 ln(dm/dt T)=-0.2–8201.4/T; r^2=0.9998; σ_a=0.1; σ_b=35.7; $\Delta_l^g H_m$(450.0 K)/kJ·mol^{-1}=68.2 ± 0.3

Series 4 ln(dm/dt T)=-0.6–8032.0/T; r^2=0.9999; σ_a=0.1; σ_b=26.3; $\Delta_l^g H_m$(450.0 K)/kJ·mol^{-1}=66.8 ± 0.2

Weighted average: <$\Delta_l^g H_m$ (1MH, 450.0 K)>/kJ·mol^{-1}= 67.4 ± 0.1

Standard uncertainties u are $u(T)$ = 0.1 K, $u(m)$ = 0.1 µg, and the combined expanded uncertainty U_c is $U_c(dm/dt)$ = 0.066·109 kg·s^{-1}, $U_c(1/T)$ = 0.001 x10^3 K^{-1}, U_c(ln(dm/dt·T)=0.020; U_c(ln(dm/dt·T)=0.020.

From the vaporization enthalpy data, the weighted average value was obtained with equation 1

$$\mu = \frac{\sum\left(\frac{x_i}{u_{comb,i}^2}\right)}{\sum\left(\frac{1}{u_{comb,i}^2}\right)} \qquad (1$$

where, x_i y $u_{comb,I}$ are the vaporization enthalpies and the combined uncertainty, respectively.

Uncertainty of final value corresponds to standard combined uncertainty, which was obtained by using equation 2.

$$u = \sqrt{\frac{N}{u_{comb,i}^2}} \qquad (2$$

Where N, is the number of experimental series.
